# Neuroprotective Natural Products for Alzheimer’s Disease

**DOI:** 10.3390/cells10061309

**Published:** 2021-05-25

**Authors:** Xin Chen, Joshua Drew, Wren Berney, Wei Lei

**Affiliations:** 1 Department of Pharmaceutical Sciences, College of Pharmacy and Health Sciences, Campbell University, Buies Creek, NC 27506, USA; jmdrew1018@email.campbell.edu (J.D.); wwberney0603@email.campbell.edu (W.B.); 2 Department of Pharmaceutical and Administrative Sciences, School of Pharmacy, Presbyterian College, Clinton, SC 29325, USA; wlei@presby.edu

**Keywords:** Alzheimer’s disease (AD), neurodegenerative, neuroprotective, natural products, antioxidant, anti-neuroinflammatory, amyloid β peptide (Aβ), neurofibrillary tangles (NFTs)

## Abstract

Alzheimer’s disease (AD) is the number one neurovegetative disease, but its treatment options are relatively few and ineffective. In efforts to discover new strategies for AD therapy, natural products have aroused interest in the research community and in the pharmaceutical industry for their neuroprotective activity, targeting different pathological mechanisms associated with AD. A wide variety of natural products from different origins have been evaluated preclinically and clinically for their neuroprotective mechanisms in preventing and attenuating the multifactorial pathologies of AD. This review mainly focuses on the possible neuroprotective mechanisms from natural products that may be beneficial in AD treatment and the natural product mixtures or extracts from different sources that have demonstrated neuroprotective activity in preclinical and/or clinical studies. It is believed that natural product mixtures or extracts containing multiple bioactive compounds that can work additively or synergistically to exhibit multiple neuroprotective mechanisms might be an effective approach in AD drug discovery.

## 1. Introduction

Alzheimer’s disease (AD), discovered by Dr. Alois Alzheimer in 1906 [1], is currently the number one chronic neurodegenerative disease, affecting more than six million people in the US and about 50 million people worldwide [2]. As a neurodegenerative disease, AD slowly and irreversibly destroys memory, cognition, and eventually the ability to carry out daily activities, leading to the need for full-time care, which is most common among people over 65, although it does occur in the younger population [3]. Age is considered the biggest risk factor for AD. About 3% of people aged 65–74, 17% of people between 75 and 84, and 32% of people 85 and over suffer from this disease [4,5]. With the size of the elderly population continually increasing, it is estimated that by the year 2050, there will be one new AD case every 33 s and the total number of AD cases will rise to 16 million in the US alone [2]. Being the most common cause of dementia among older adults and listed by the Centers for Disease Control and Prevention (CDC) as the sixth leading cause of death in the US, AD may actually be ranked third, just after heart disease and cancer, in major causes of death for older people [6].

In contrast to the high prevalence of AD, we only have five drugs approved by the FDA for its treatment, namely rivastigmine, galantamine, donepezil, memantine, and memantine in combination with donepezil. Furthermore, none of these drugs can reverse, stop, or even slow down the damage and destruction of neurons that cause AD symptoms and make the disease fatal. These largely unmet medical needs, together with the physical, emotional, societal, and healthcare-related impacts of AD, have challenged the research community to better understand and come up with more effective ways to manage this formidable disease. Enormous efforts have been devoted to the identification of the underlying mechanisms of this disease and the discovery of disease-modifying therapies that can halt the progression of AD [7].

Although the exact biological cause of AD in most people is still not fully understood, the hallmarks of this disease are believed to be the abnormal deposition of insoluble β-amyloid peptide (Aβ) and the accumulation of neurofibrillary tangles (NFTs) of phosphorylated tau protein in neuronal cytoplasm, which results in atrophy and neuron death [8]. Several biological processes have been linked to these neurodegenerative changes including neuroinflammation, depletion or insufficient synthesis of neurotransmitters, oxidative stress, collapse of calcium homeostasis, and abnormal ubiquitination [9]. As a result, neurons and synapses participating in memory processes are damaged, leading to a decline in learning ability and other cognitive functions. All the currently available medications for AD, except for memantine which blocks NMDA receptors in the brain from excess stimulation that can damage nerve cells, increase the amount of neurotransmitters in the brain to temporarily improve cognitive symptoms. These drugs’ effectiveness varies a lot individually and is limited in duration. Other approaches, including targeting Aβ plaques [10] and tau NFT aggregations [11], reducing oxidative stress [12] and neuroinflammation [13], etc., have been attempted, with a number of new drug candidates being evaluated in preclinical and clinical studies.

Natural sources, including plants, animals, microbes, and the marine world, provide abundant bioactive compounds with complex structures and novel pharmacological properties [14]. As one of the major sources of drug discovery, natural products and their isolated compounds have been extensively studied in efforts to develop more effective drugs for the management of AD [15]. In fact, the cholinesterase inhibitor galantamine is a natural product itself [16] and rivastigmine is a semi-synthetic derivative of a natural product called physostigmine [17]. Mixtures or extracts of natural products might have advantages compared to individual natural compounds, as they have multiple simultaneous target approaches, which could be a novel treatment option for AD, considering the complexity of its pathophysiology [18]. Mounting evidence has suggested that herbs or herbal formulations, together with mixtures obtained from other natural sources, may provide cognitive benefits to AD patients [15]. Consequently, various natural sources and their extracts are extensively employed in animal models and AD patients [19,20].

This review focuses on the natural product extracts or mixtures that may have neuroprotective effects through various mechanisms for the prevention and treatment of AD. The systematic literature search was conducted using SciFinder and PubMed as online databases until February 2021 with key words being neuroprotective natural products and AD. Among all the literature meeting our criteria, we especially focused on natural product extracts and mixtures.

## 2. Neuroprotective Mechanisms of Natural Products for AD

### 2.1. Overview of the Mechanisms Underlying AD

As an age-related progressive neurodegenerative disease accounting for most dementia cases, the intricate pathogenic mechanisms of AD are not fully understood. Besides the genetic and environmental factors which are believed to contribute to the etiology of AD, several hypotheses have been proposed to explain this complicated disorder with the most prevalent ones being the Aβ cascade hypothesis, tau hypothesis, inflammation hypothesis, cholinergic hypothesis, and oxidative hypothesis [21]. According to the Aβ cascade hypothesis, Aβ peptides are the causative agent in AD because the extracellular deposition of Aβ peptides as senile plaques (SP) and NFTs will result in neuron loss, vascular damage, and dementia [22]. Considered as another intracellular hallmark of AD, NFTs mainly consist of tau protein, which is a microtubule-associated scaffold protein enriched in the axons of neurons. Its aggregation impairs axons, causing neurodegeneration [23]. Recently, the inflammation hypothesis has emerged as the next major pathology in AD, involving the sustained immune response in the brain. Continued activation of the brain’s immune cells such as microglia results in the production and release of numerous proinflammatory cytokines, which not only leads to neuronal loss, but also facilitates both Aβ and tau pathologies [24]. Cholinergic neuronal damage has been widely accepted as a major pathological change correlating with the cognitive impairment in AD patients. Therefore, the cholinergic hypothesis suggests that a dysfunction of cholinergic neurons in the brain contributes substantially to the cognitive decline in AD [25]. This hypothesis is supported by the use of cholinesterase inhibitors in AD treatment. Oxidative stress has also been discovered to play an essential role in AD pathogenesis. Direct supporting evidence has indicated that AD is always accompanied with high cellular oxidative stress in the brain, caused by the increased generation of free radicals, increased lipid peroxidation and decreased polyunsaturated fatty acid, increased protein and DNA oxidation, as well as the accumulation and aggregation of Aβ, which also induces oxidative stress [26].

### 2.2. Neuroprotective Strategies for AD

With the substantial amount of evidence indicating that the primary causative factor in the pathogenesis of AD is the accumulation of Aβ [27], decreasing Aβ has become the major strategy in developing new therapeutics for AD [28]. However, successful AD therapeutic regimens may require multiple neuroprotective agents being used concomitantly. Through careful examination of the pathophysiological processes occurring in AD, several molecular targets have been identified as mediating these processes. These targets could aid in the development of potentially high-yield neuroprotective strategies [29]. Possible neuroprotective mechanisms focus on the inhibition of deleterious intraneuronal mechanisms triggered by Aβ and other toxic stimuli through specific interaction with various neuronal targets [30]. Practical neuroprotective approaches for managing AD include the discovery of small molecules to block Aβ interactions with its extracellular and intracellular targets [31], to minimize stress kinase signaling cascades [32], to prevent caspase activation [33] and pro-apoptotic protein expression [34], to inhibit excessive tau protein phosphorylation [35], to counteract cholinergic function loss [36], to promote the trophic state and neuron plasticity [37], to hinder reactive oxygen species accumulation [38], to suppress neuroinflammation [39] and to block excitotoxicity [40]. It is worth mentioning that some of the neuroprotective agents exhibit their effects through more than one approach. This is especially true with mixtures and extracts of natural products that contain more than one bioactive compound. Therefore, the neuroprotective effects from mixtures and extracts of natural products are always multidimensional and offer an advantage for the treatment of AD compared to single compound. Furthermore, the additive or synergistic action of crude extracts or mixtures can eliminate some of the side effects associated with the predominance of a single xenobiotic compound, providing a more comprehensive spectrum of activity, and minimizing the chances of pathogens developing resistance [41]

### 2.3. Neuroprotective Effects from Natural Products

**Figure 1:**
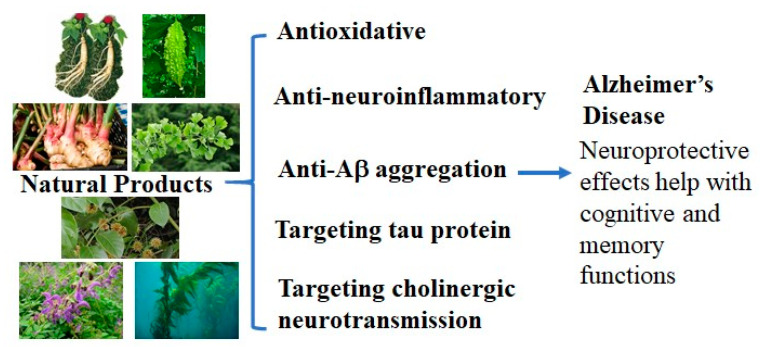
Neuroprotective effects from natural products for AD.

Natural products have been shown to play neuroprotective roles through almost all of the different molecular mechanisms mentioned above (Figure 1). When focusing on the mixtures and extracts of natural products, the observed neuroprotective effects have typically been recognized as being obtained through anti-oxidative or anti-neuroinflammatory activities, preventing the aggregation of Aβ and tau protein as well as through enhancing cholinergic signaling. It is reasonable to speculate that the onset and progression of AD could be slowed down or even prevented by natural products working on multiple pathological targets [42].

#### 2.3.1. Anti-Oxidative Neuroprotective Activity from Natural Products for AD

As molecules with radical and non-radical oxygen species, reactive oxygen species (ROS) and reactive nitrogen species (RNS) are highly chemically reactive. Maintaining a moderate concentration of these oxidative species is believed to play a crucial role in a number of physiological processes inside the human body, such as regulating the cell cycle, activating enzymes and receptors, as well as monitoring inflammation, phagocytosis, gene expression, and signal transduction [43,44,45,46]. The human body can neutralize these oxidative species and eliminate them so that their concentrations remain within the normal range. One of the major approaches includes the nuclear factor E2-related factor 2 (Nrf2) pathway. Nrf2 is a transcription factor that activates the expression of antioxidant genes in response to oxidative stress. A common response element of all these antioxidant genes is called antioxidant response element (ARE), which is highly involved in reducing oxidative stress, inflammation, and the accumulation of toxic metabolites [47]. However, when there is an imbalance between the production/accumulation of these oxidative species and their neutralization/elimination, the over-limitation concentrations of ROS and RNS can result in oxidative stress with serious pathological damage [48]. Specifically, because of the increased metabolic activity and limited cellular regeneration, oxidative stress shows more significant effects in the brain compared with other parts of the body [49]. In AD, Nrf2 expression is found to be upregulated in the neurons because of oxidative damage. At the same time, the levels of some ARE-containing gene products are reduced, indicating the disruption of this pathway [47]. Additionally, in vivo studies have also implied that inhibiting Keap1, which is the negative regulator of Nrf2, could prevent the Aβ_42_-mediated neurotoxicity that initiates AD [50]. Consequently, oxidative stress is widely accepted as playing a major role in progressively damaging neuron structure and impairing neuron function, which is considered one of the major causes of the development of severe neurodegenerative disorders, including AD [44]. Therefore, tremendous research efforts have been devoted to developing antioxidant therapies as neuroprotective agents for the treatment of AD [51].

Antioxidants are compounds that can react with free radicals to convert them into harmless species. Extensive research has identified oxidative stress as a primary factor in the development and progression of AD, and antioxidants as being capable of counteracting the damaging effects of oxidation [52]. Based on their occurrence in nature, antioxidants are grouped into two categories, natural and synthetic antioxidants, with most synthetic antioxidants being derived from natural products. The most well-known natural antioxidants are β-carotenoid (vitamin A), ascorbic acid (vitamin C), α-tocopherol (vitamin E), carotenoids, and flavonoids, all of which play an important role against ROS-induced damage in an organism.

Many of the natural antioxidants are obtained from plants and structurally belong to the classes of compounds called phenolics and polyphenolics, as well as carotenoids and antioxidant vitamins, among others. Phenolics and polyphenolics, which have one or more hydroxyl groups on their aromatic ring(s), have been well established as possessing high antioxidant capacity because of their capability to scavenge free radicals, donate hydrogen atoms and electrons, and chelate with metal cations [53]. The structures of these phenolics and polyphenolics, especially the hydroxyl groups substituted on the aromatic rings give them the capacity to perform their antioxidative function [54]. Being the most common group of polyphenolic compounds, flavonoids exhibit a wide range of antioxidative actions against free radical-mediated cell signaling, inflammation, and tumors [55]. As AD may be caused by this impaired cell signaling, it is not surprising that some of the natural flavonoids, derivatives of flavonoids, and natural sources rich in flavonoids are being extensively evaluated for the treatment of AD [56]. The neuroprotective effect of flavonoids is partially attributed to their antioxidative properties that can prevent free radical formation by modulating the cell signaling pathways involved in antioxidative protein expression, glutathione synthesis, as well as cell proliferation and survival [57].

Non-flavonoids, which is the other group of phenolic compounds, have slightly more variable structures compared to flavonoids. Some of the non-flavonoids such as phenolic acids, tannins, lignans, stilbenes, quinones, coumarins, and curcuminoids also exhibit high antioxidant activities, especially phenolic acids [58]. Dietary polyphenols have been demonstrated to prevent neuron damage and apoptosis in vitro and in vivo through attenuating ROS levels as a major mechanism for reducing the oxidative stress involved in the onset and progression of AD [59]. Some phenolic acid compounds isolated from wine have shown neuroprotective effects in vitro, partially via the prevention of RNS-induced stress injury [60]. Phenolic acids isolated from the medicinal plant *Rosmarinus officinalis* have exhibited neuroprotective activity in vitro through decreasing ROS/RNS levels and silencing Nrf2 expression [61]. As phenolic compounds are naturally occurring substances found in integral parts of the human diet like fruit, vegetables, nuts, seeds, flowers, and some herbal beverages, their dietary intake has been shown to be able to lower the incidence of chronic degenerative diseases, including AD. Phenolic compounds and their natural sources have received considerable attention recently for their antioxidant capacities as neuroprotective agents for better management of AD [51].

Carotenoids are another group of natural products that can interact with free radicals and exhibit antioxidant properties [62]. As lipophilic pigmented compounds primarily found in plants, algae, as well as in microorganisms including yeasts, fungi, archaea, and eubacteria, carotenoids have an isoprenoid skeleton which is folded and connected in a variety of ways to form numerous different structures possessing different physicochemical and biological properties, along with yellow, orange, or red colors. Their structures comprise conjugated double bonds within a long polyenic carbon chain and a nearly bilateral symmetry around the core, which allows electrons to move freely across the molecule and provides the capability to interact with free radicals to offer antioxidative activity [63]. Hence, when consumed in adequate levels, carotenoids have been demonstrated to bring many health benefits to the human body, especially the prevention of AD symptoms, partially through the reduction of ROS/RNS.

In fact, it has been found that AD patients have significantly lower serum levels of six measured carotenoids, along with other antioxidants, such as retinol and α-tocopherol. Moreover, antioxidants, including vitamins and carotenoids, may confer a reduction in oxidative damage as a neuroprotective defense mechanism in AD patients [64]. Dietary supplementation of carotenoids has been discovered to ameliorate AD symptoms in AD animal models [65]. The beneficial effects of consuming carotenoids such as lutein and zeaxanthin on reducing AD incidence was further supported by a recent clinical study [66]. A number of epidemiological studies have also connected the consumption of a carotenoid-rich diet with a decreased risk of neurodegenerative diseases, including AD [67].

#### 2.3.2. Anti-Neuroinflammatory Neuroprotective Activity from Natural Products for AD

Although the exact pathophysiological mechanism of AD has not been elucidated yet, various possible mechanisms have been proposed to explain this multifactorial disorder including the Aβ hypothesis, tau hypothesis, cholinergic hypothesis, and inflammation hypothesis [68]. Neuroinflammation has been indicated to be associated with the deposit of Aβ in the brain, which is a major hallmark in the pathology of AD [69]. The neuroinflammatory process of AD results from elevated level of ROS, increased microglial activation, production of cytokines, and activated nuclear factor kappa B (NF-κB) [70]. Specifically, the activation of immune cells like microglia leads to the production and secretion of proinflammatory cytokines, including IFN-γ, IL-1β, and TNF-α [71], which stimulate the astrocytes nearby to generate Aβ_42_ oligomers [69]. Extracellular insoluble Aβ aggregates can attract more microglial cells to form microglia clusters at the Aβ deposition site [72]. These pro-inflammatory cytokines were found at elevated levels in the brain, serum, and cerebrospinal fluid of AD patients [72]. Countless studies have directly correlated the cognitive decline in AD patients with the increased levels of cytokines at all stages of the disease [73]. Best characterized as a transcription factor ubiquitously expressed to regulate the expression of many genes, NF-κB controls the encoding of proteins participating in the inflammatory and immunity processes [74]. Additionally, it is also directly involved in brain function, especially in neurodegenerative diseases like AD. The neuroprotective mechanism resulting from the activation of the Nrf2 pathway has also been correlated to the anti-inflammatory effects involving NF-κB [75] as NF-κB is a well-known negative regulator of Nrf2 [76]. Its activation has been shown to be linked with Aβ−induced neurotoxicity [77] and can be detected in the brains of AD patients [78]. Long-term usage of anti-inflammatory drugs can suppress the onset and progression of AD, indicating NF-κB as a key mediator of brain inflammation in AD [79].

The neuroprotective potential of natural products stemming from their anti-inflammatory properties, together with their antioxidant activities, has uncovered the potential to prevent and improve neurodegeneration in AD with minimal side effects compared to synthetic drugs [80]. As inflammation can also contribute to neurodegeneration and accelerate the progression of AD, natural products with anti-inflammatory properties might serve as a potential treatment to reduce the symptoms of AD, not only in the early prevention stage, but also in the disease management stage [80]. The effects of these natural products with anti-inflammatory activity are believed to have a synergistic effect involving interaction with multiple targets and modulation of multiple signaling pathways. Natural products, with their capacity to inhibit neuroinflammation, are able to produce anti-amyloid effects as a beneficial outcome in the management of AD [81]. Therefore, it is worthwhile to evaluate natural products or mixtures of natural products for their multi-target anti-inflammatory activity as potential therapeutic candidates for the prevention or management of AD. In fact, some plant-based and animal-based natural products, such as omega-3 fatty acids, have exhibited neuroprotective efficacy through their anti-inflammatory effects [82].

Several mechanisms have been reported for the anti-neuroinflammatory activity of some natural products, including the inhibition of microglia activation, the reduction of pro-inflammatory cytokine release from activated microglia, the inhibition of NF-κB, and the activation of p38 MAPK. The activity of those natural products that can activate Nrf2 and possess antioxidant properties is also at least partially attributed to their anti-neuroinflammatory activity [75].

Natural products with anti-neuroinflammatory activity can be found in almost all major structural categories like alkaloids, polyphenols, terpenes, carotenoids, and marine natural products. Alkaloids are nitrogen-containing (usually inside a ring structure) bioactive secondary metabolites with a wide variety of pharmacological activities, including anti-inflammatory effects. One example of an alkaloid with anti-neuroinflammatory activity is cryptolepine, obtained from *Cryptolepis sanguinolenta*. It has been reported that cryptolepine can reduce the levels of TNFα, IL-6, IL-1β, NO, and PGE2 in LPS-stimulated rat microglia via the blockage of the activation of NF-κB, p38 MAPK in the microglia [83]. Similarly, another alkaloid isolated from *Radix Stephania tetrandra* named tetrandrine shows promising anti-neuroinflammatory activity through the inhibition of NF-κB activation in a rat model of AD [84]. Flavonoids and other polyphenolic compounds have been extensively described to have anti-inflammatory activity. Examples in this structural group include kaempferol [85], tiliroside [86], apigenin [87], quercetin [88], epigallocatechin-3-gallate (EGCG) [89], punicalagin [90], urolithin A [91], mangiferin [92], resveratrol [93], curcumin [94], and many more. Their anti-inflammatory activity mostly involves the reduced production of pro-inflammatory mediators and the inhibition of activation of NF-κB and p38 MAPK pathways [95]. Within this group, flavonoids are considered as the most important subgroup for inhibiting neuroinflammation in AD because of their fundamental inhibitory actions on pro-inflammatory transcription factors, in addition to their capability of activating antioxidant/anti-inflammatory transcription factors. Terpenoids, having two or more isoprene units in their structures, have also been widely reported on for their anti-neuroinflammation properties in vitro and in animal models. Parthenolide, which is a sesquiterpene lactone present in *Tanacetum parthenium* [96], artemisinin, that is another sesquiterpene lactone found *Artemisia annua* [97], thymoquinone, which is the bioactive constituent of *Nigella sativa*, carnosic acid and carnosol, that are natural diterpenes found in *Rosmarinus officinalis* [98], and *Ginkgo biloba* extract, that is rich in polyphenolic compounds and terpene lactones [99], have all been demonstrated to inhibit neuroinflammation by reducing levels of pro-inflammatory mediators and regulating Nrf2, NF-κB and p38 MAPK pathways. Although a spice, *Crocus sativus* (Saffron) is famous for its wide variety of therapeutic applications, including AD [100]. Carotenoids obtained from *Crocus sativus* are believed to have anti-neuroinflammatory activity on cognitively impaired mice through the mechanism related to NF-κB [101]. Astaxanthin, a xanthophyll carotenoid found in *Haematococcus pluvialis*, *Chlorella zofingiensis*, *Chlorococcum*, and *Phaffia rhodozyma*, is probably the most intensively investigated marine natural product with neuroprotective potential. In BV-2 microglia, it was able to inhibit NO/iNOS and COX-2 [102], and attenuate the production of IL-6 induced by LPS through the activation of ERK1/2-MSK1 and NF-κB [103].

#### 2.3.3. Anti-Aβ Aggregation Neuroprotective Activity from Natural Sources for AD

Produced through the proteolytic process of Aβ amyloid precursor protein (APP), a transmembrane protein, by β- and γ-secretases, Aβ is hypothesized to be the main cause of AD. Its accumulation in the brain is considered to be an early toxic event in AD pathogenesis that can cause memory loss and eventually lead to personality changes and cognitive decline over time [104]. The most toxic species of Aβ aggregates is believed to be the small, soluble aggregates called oligomers, which play an essential role in cell and tissue toxicity, especially in neurodegenerative diseases like AD [105]. While the exact toxicity mechanisms of amyloid oligomers remain unclear, results from several in vitro and in vivo studies revealed that high levels of amyloid oligomers were capable of over-stimulating the glutamatergic synaptic transmission and causing synapse loss [106]. They could also interact aberrantly with the cell membrane [105] and are reported to be associated with oxidative stress [107], inflammation [108], mitochondrial dysfunction [109], and disturbed calcium homeostasis, which is ultrasensitive to any changes in membrane permeability [110]. The amyloid cascade hypothesis of AD, which incorporates genetic, biochemical, and histological evidence, posits that the deposition of Aβ in the brain, due to an imbalance between its production and clearance, initiates a sequence of events that ultimately leads to AD dementia. The amyloid cascade hypothesis of AD proposes that Aβ aggregation is the primary event, and that aggregation of tau, inflammation and other changes observed in AD brains are downstream. Prohibiting the formation of Aβ aggregates, as well as reducing or removing them should be therapeutically useful in AD treatment.

Generally, the level of Aβ is determined by the rates of its production and removal through proteolytic degradation. Hence, any defects in the formation and degradation of Aβ may also affect its level and potentially contribute to AD. As the most prevalent mechanism in AD pathogenesis, the Aβ hypothesis indicates that extracellular deposits of Aβ form SP and NFTs, leading to neuronal loss and vascular damage [111]. The major proteinaceous Aβ peptide found in plaques of AD exists in the Aβ_1–40_ and Aβ_1–42_ forms, which are found to exist as monomers but in rapid equilibrium with the corresponding soluble oligomers through self-assembly [112].

One of the major focuses of drug discovery efforts in the AD area has been the development of drug candidates to regulate abnormal Aβ aggregation. Several synthetic drugs have been evaluated in clinical trials, including LY2886721 [113], AN1792 [114] and verubecestat [115], along with monoclonal antibody drugs such as donanemab, which targets Aβ_3–42_ [116] and aducanumab (BIIB037), which targets a conformational epitope found on Aβ [117]. However, none of them have been successful in the treatment of AD. Recently, rigorous investigation has been focused on exploring natural products for safer and inexpensive therapeutics, which can provide a better solution for managing AD [118]. There are two different major approaches in the development of such natural product-based AD therapies: targeting the secretases to stop the formation of Aβ, and interfering directly with Aβ aggregates [119].

Aβ peptides are formed after APPs get cleaved by secretases. After being synthesized in the brain, APPs translocate to the plasma membrane where they undergo specific endoproteolytic cleavages by α-, β-, and γ- secretases via either amyloidogenic or non-amyloidogenic pathways [120]. In the non-amyloidogenic pathway, α-secretase is the first enzyme to cleave APPs and generate the soluble N-terminal fragment (sAPPα) that is released into the extracellular space, and the membrane-tethered C terminal fragment CTFα which is further cleaved by γ-secretase to produce the extracellular P3 peptide and the APP intracellular domain (AICD) that is released into the cytosol [120]. In the amyloidogenic pathway, β-secretase, or beta-site amyloid precursor protein cleaving enzyme (BACE), cleaves APPs to create soluble APPβ fragment, which goes into the extracellular space, and leaves the rest of the protein (CTFβ) on the plasma membrane, which is further cleaved by γ-secretase to yield Aβ peptides with 40–43 amino acids [121]. In the non-amyloidogenic pathway, the cleavage of APPs by α-secretase first leads to the production of intracellular AICD fragments and extracellular P3 peptide with no Aβ deposition. However, in the amyloidogenic pathway, BACE cleaving APPs eventually results in the generation of Aβ peptides. Two major types of BACE, BACE1 and BACE2, have been found with similar structures. Because α- and β-secretases need to compete for the process of APP cleavage, and the cleavage of APP by α-secretase prevents the Aβ formation, two strategies have become very promising in developing drugs targeting these two secretases to overturn the neuropathological changes linked with most AD symptoms: increasing the activity of α-secretase or decreasing the activity of β-secretase [122].

Several natural compounds have demonstrated their capacity to regulate Aβ production in AD by modulating the activity of α- or β-secretase. As triterpene saponin (glycoside) components obtained from the root of ginseng (*Panax ginseng*), ginsenoside Rg1 can increase α-secretase level and decrease β-secretase level in vitro [123]. It can also significantly reduce the Aβ level in the cerebra of transgenic AD mice and improve the cognitive deficit of the mice [124]. From *Glycyrrhiza glabra*, a phenolic compound 2,2′,4′-trihydroxychalcone (TDC) has been isolated and has shown neuroprotective effects both in vitro and in vivo through decreasing BACE1 levels without affecting α- or γ-secretase levels [125]. Another phenolic compound, hispidin, extracted from the fungus *Phellinus linteus*, has been discovered to inhibit BACE1 in vitro without affecting the activities of α- and γ-secretases [126]. The polyphenol compound EGCG, most abundantly derived from green tea, has been proven to increase α-secretase secretion, and prevent abnormal Aβ aggregate formation in vitro [127]. At the same time, it can reduce the activity of β-secretase and inhibit the amyloidogenic pathway [128]. Extracted from black ginger or citrus peels and widely used for allergy and viral infections, polymethoxyflavones can reduce the enzymatic function of BACE1 in the amyloidogenic pathway while not affecting the activities of α-secretase as its mechanism for inhibiting BACE1-related amyloidogenesis in vitro [129]. As an isoquinoline alkaloid extracted from *Coptidis rhizome* and *Berberis vulgaris*, berberine could reduce BACE1 activity, decrease Aβ level, and improve behavioral symptoms in AD animal models via inhibiting β/γ-secretases activity and enhancing α-secretases [130]. Ligustilide, derived from *Ligusticum chuanxiong*, was able to promote the non-amyloidogenic pathway of APP cleavage in vitro and in vivo through increasing the activity of α-secretase [131].

During the process of Aβ peptide aggregation, phenylalanine 19 (F19) and phenylalanine 20 (F20) residues in the hydrophobic core domain are believed to be critical for the hydrophobic interactions among β-strands, which is thought to be the primary driving force for Aβ aggregation [132]. Therefore, targeting F19 and F20 residues of Aβ might be able to alter the structure of Aβ aggregates and decrease the related toxicity. Several natural products have been reported to be able to target these two phenylalanine residues and interact with Aβ through hydrogen bonds or hydrophobic interactions to destroy the normal structure of Aβ fibrils and block its toxicity in AD. Examples of these natural products include the polyphenol stilbene compound resveratrol that can decrease the formation of Aβ plaques [133], the major compound from the Sappan wood brazilin that can prevent abnormal Aβ aggregation [134], the polyphenol compound curcumin that interacts with F19 and F20 in the hydrophobic core domain of Aβ and disrupts their β-sheet structure to alleviate their toxicity [135], a polyphenol compound tannic acid that can stack with the hydrophobic domain of Aβ to prevent their polymerization into fibrils [136], destabilize the conformation of Aβ fibrils and promote the depolymerization of Aβ [137], and a group of polycyclic polyphenols theaflavin that binds to the hydrophobic core domain and hydrophobic C-terminus of Aβ to prevent the formation of toxic Aβ aggregates and induce the remodeling of misfolded Aβ aggregates [138].

#### 2.3.4. Neuroprotective Activity Targeting Tau Protein from Natural Products for AD

The microtubule-associated protein tau is essentially disordered because of its high flexibility and lack of a stable conformation. One major hallmark of AD is the accumulation of NFTs of abnormal tau in neuronal cytoplasm. Therefore, tau needs to be detached from microtubules and then transferred into abnormal aggregates before the onset of AD occurs. This process is believed to be caused by a series of post-translational modifications such as phosphorylation, acetylation, ubiquitination, glycosylation, nitration, methylation, and so forth, with phosphorylation being the most important modification [139]. In fact, tau protein is hyperphosphorylated in the brain in AD, with 3–4 times more phosphorylation compared to a healthy brain [140]. Tau hyperphosphorylation promotes the dissociation of tau from microtubules and induces pathological tau aggregation [141]. Other abnormal post-translational modifications of tau, including acetylation [142], ubiquitination [143], methylation [144], glycation [145], etc., have also been identified in AD. Based on our knowledge about tau pathology in AD, several potential approaches have been proposed to block tau-mediated neurotoxicity, which mostly include inhibition of tau post-translational modifications and direct inhibition of tau aggregation.

Tau dephosphorylation is mainly carried out by protein phosphatase 2A (PP2A), which has reduced activity in the AD brain and cannot be easily targeted by drugs [146]. Subsequently, protein kinase inhibitors are usually developed with the intention to target other kinases to inhibit tau hyperphosphorylation or reduce tau aggregation rather than directly target PP2A [147]. Some natural drugs have been shown to inhibit tau hyperphosphorylation in AD animal models through modulating the activity of cyclin-dependent kinase-5 (CDK5) [148], glycogen synthase kinase-3 (GSK3) [149], or PP2A [150] to improve the symptoms of AD. Tongmai Yizhi Decoction, which contains six raw materials together with huperzine A, significantly decreases CDK5 and CDK5 expression in the hippocampus of model rats [151]. Safflower yellow from *Carthamus tinctorius* (Asteraceae) inhibits the GSK-3 activation and GSK-5 signaling pathways to prevent tau hyperphosphorylation by Aβ_1–42_ and improves learning and memory functions in AD model rats [148]. Geniposide isolated from the fruit of *Gardenia jasminoides* (Rubiaceae) reduces the hyperactivity of GSK3β induced by STZ and improves the spatial learning of rats [152]. Ginsenoside Rd from *Panax ginseng* increases PP2A activity and decreases okadaic acid-induced neurotoxicity, as well as tau hyperphosphorylation in vitro and in vivo [149].

Many of the tau aggregation inhibitors are natural products with antioxidant properties. Crocin from *Crocus sativus* (Iridaceae) can interfere with tau protein nucleation and inhibit tau protein filament formation in vitro [150]. Four simple quinones (1,4-benzoquinone, 1,4-naphthoquinone, 9,10-anthraquinone and 9,10-phenanthraquinone) from food or medicinal plants and four anthraquinone compounds (chrysophanol, emodin, aloe-emodin, and rhein) from *Rheum rhabarbarum* (Polygonaceae) have been found to inhibit the formation of toxic insulin oligomers in vitro, which may be helpful in preventing protein misfolding diseases like AD [153]. In vitro, the extracts of *Glycyrrhiza inflata* (Fabaceae) and *P. ginseng* (Araliaceae) are able to improve the growth of the repeat domain and axons in mutant tau protein to prevent tau aggregation. The aqueous extract of *G. inflata* (Fabaceae) can further upregulate unfolded protein response-mediated chaperones to reduce tau misfolding [154]. Another in vitro study has revealed that α-cyperone from rhizomes of *Cyperus rotundus* (Cyperaceae) exerts a significant effect on reducing the rate of tubulin polymerization and the concentration of polymerized tubulin, which may represent a beneficial neuroprotective strategy for AD [155]. Other well-known examples of natural products capable of inhibiting tau protein aggregation are curcumin, which inhibits amyloidogenic protein aggregation including Aβ and tau [156], resveratrol, which inhibits the aggregation of the repeat domain of tau along with many other neuroprotective mechanisms [157], purpurin, which inhibits tau fibrillization and breaks down the pre-formed fibrils [158], folic acid, which inhibits tau aggregation via stabilizing its native state [159], and the root extract of red ginseng [160].

#### 2.3.5. Neuroprotective Activity Targeting Cholinergic Neurotransmission from Natural Sources for AD

The cholinergic system, which uses acetylcholine (ACh) as a neurotransmitter, is associated with a number of cognitive functions including learning and memory. The cholinergic hypothesis of AD proposes that the loss of these neurons in the brain is associated with cognitive deficits [161]. In fact, three out of the four approved single drugs work by increasing the lifespan of ACh by inhibiting the enzyme acetylcholinesterase (AChE), which is mainly responsible for metabolizing Ach, and has been recognized as the most effective drug target for developing AD drugs [162]. Other strategies targeting cholinergic neurons and acetylcholine to protect from AD-related neurotoxicity include promoting the expression of choline acetyltransferase (ChAT), which is a key enzyme in the acetylcholine biosynthetic pathway, and protecting cholinergic neurons by stimulating the expression of nerve growth factor (NGF), brain-derived neurotrophic factor (BDNF), and their receptors [163].

In a study with aqueous extracts from 80 traditional Chinese medicinal plants, *Berberis bealei Fortune* (Berberidaceae), *Coptis chinensis Franch* (Ranunculaceae) and *Phellodendron chinensis* (Rutaceae), all of which contain large amounts of isoquinoline alkaloids, were shown to effectively inhibit AChE function in vitro. Synergistically enhanced inhibitory activity has been found with the alkaloid combinations of berberine, coptisine, and palmatine [164]. Extracts from *Huperzia serrata* (Lycopodiaceae) inhibited AChE activity and ameliorated the cognitive impairment of AD mice [165]. A number of lycopodium alkaloids have been isolated and synthesized as potent AChE inhibitors [166]. Extracts from *Crocus sativus* (Iridaceae) exhibited moderate inhibitory activity against AChE. Crocetin, dimethylcrocetin, and safranal have all been found to possess moderate AChE inhibitory activities with IC_50_ values below or around 100 µM [167]. *Gastrodia elata* (Orchidaceae) could substantially increase ChAT expression in the medial septum and hippocampus to improve spatial memory in AD mice [168]. Compound Danshen Tablet increased ChAT expression in the brain, induced BDNF production, and activated the PKC receptor to improve spatial recognition in an AD rat model [169]. A variety of traditional Chinese medicine extracts demonstrated excellent therapeutic effects on AD through their effects on the expression of NGF, BDNF, and their related receptors in vivo [170]. The most famous ones are Bushen-Yizhi formula that could regulate NGF signal transduction and the anti-apoptotic cholinergic pathway to improve the IBO-induced memory impairment in an AD rat model [170], a bioactive components of ginger 6-shogaol that could increase the NGF levels of NGF and improve Aβ or scopolamine-induced memory impairment in animal models of dementia [171], *Xanthoceras sorbifolium* (Sapindaceae) extracts that could protect dendritic spines through the BDNF signal transduction pathway and improve cognition in an AD rat model [172], tanshinone IIA that could promote depolarization-induced BDNF synthesis [172], and polygonum multiflorum Thunberg complex composition-12 (PMC-12) that could increase BDNF level and synapse number in the hippocampus of an AD mice model [173].

## 3. Neuroprotective Natural Products for AD

When going through all the possible neuroprotective mechanisms that natural products may have to treat AD, it is clear that most of the individual natural compounds may exert their neuroprotective activity through more than one mechanism. One excellent example is the polyphenol flavonoid resveratrol whose neuroprotective potential consists of antioxidant activity [174], anti-neuroinflammatory activity [175], promoting the clearance of Aβ peptides [175], inhibition of GSK3β, and decreased brain levels of phosphorylated tau [176], as well as increasing cholinergic neurotransmission [177] and BDNF expression [178]. On the other hand, natural product mixtures or extracts, containing numerous individual compounds, may possess better neuroprotective potential as some of these compounds can work synergistically to present more profound effects on preventing neurotoxicity [164]. Moreover, extracts or mixtures of natural products directly obtained from their origins are more affordable therapeutic options with fewer side effects. In fact, some of these natural product mixtures or extracts have shown very promising neuroprotective activities in vitro and in vivo with quite a few being evaluated in clinical trials for AD right now. Here, we summarize the information about natural product mixtures or extracts with their neuroprotective activities for AD.

### 3.1. Neuroprotective Natural Products from Medicinal Plants for AD

Medicinal plants have been discovered to be able to decrease AD progress and symptoms [179]. Both the extracts and the active individual compounds from medicinal plants have been intensively investigated for their effects on AD [180]. The active compounds isolated from medicinal plants, such as phenolic lignans, flavonoids, tannins, and polyphenols, as well as triterpenes, sterols, and alkaloids, have exhibited various beneficial neuroprotective functions, including antioxidant, anti-neuroinflammatory, anti-amyloidogenic, anti-tau aggregation, and anticholinesterase activities [179]. Some of these active compounds, either as single components like curcumin, melatonin, resveratrol, and vitamins C and E, or as herbal extracts such as aged garlic extract, *Ginkgo biloba* extract, and green tea have been evaluated in AD patients with positive results [181]. Below, the information on the herbal extracts from medicinal plants that show neuroprotective effects for AD is summarized.

#### 3.1.1. Pistacia Genus

Plants in the genus Pistacia are probably among the most precious natural resources with neuroprotective potential based upon their traditional applications. Related literature has shown that the neuroprotective effects of the genus Pistacia mainly include antioxidant, anti-neuroinflammatory, anti-Aβ aggregation, AChE inhibitory activity, and regulation of some other cellular pathways [182]. The kernel extract of *Pistacia vera* could inhibit cisplatin or vincristine-induced cognitive and motor impairments in vivo, which might be related to its high flavonoid and phenolic content [183]. Its seed oil has also been reported to improve memory and cognitive impairment in vivo [184]. The hydroalcoholic extract of pistachio nuts significantly improved learning and memory in the AD rat model [185]. The aqueous methanolic extract of its hull could inhibit AChE in vitro [186]. The hexane extract of the pistachio nuts showed antioxidant activity in vitro [182]. The gum extract also exhibited antioxidant neuroprotective effects in rats [182].

Pretreatment of the essential oil from the fruit of *Pistacia lentiscus* was reported to attenuate lipopolysaccharide-induced memory impairment in rats, decrease AChE activity and oxidative stress markers in brain tissue with the major components being 4-(3-[(2hydroxybenzoyl)amino] aniline)-4-oxobut-2-enoic acid, β-myrcene, 3-pentadecylphenol, P-tolyl ester, aminoformic acid, and β-sitosterol [187]. The aqueous extract of its leaves and the ethanolic extract of its oleoresin also possessed AChE inhibitory activity with IC_50_ values around 10 μg/mL [188]. Moreover, its dichloromethane extract oleoresin has also shown AChE inhibitory activity in vitro [189]. The methanolic extract could reduce Aβ-mediated cellular toxicity in SH-SY5Y cells [182]. The alcoholic extract from its leaves has also been shown to prevent oxidative damage-induced disorders, thanks to its substantial phenolic content, as well as significantly protect neuron cells against oxidative injury by Aβ_25–35_ and H_2_O_2_. This could almost completely protect the cells against Aβ-induced neurotoxicity [190]. The essential oil from the leaves has been demonstrated in multiple studies to possess anti-neuroinflammatory activity in vivo through various mechanisms such as inhibiting COX2 and stimulating PPAR-α etc. [191]. Taken together, this plant exerts neuroprotective effects which might be helpful in preventing and treating AD symptoms.

The ethyl acetate and aqueous extracts from the leaves of *Pistacia atlantica* have been described to have effective AChE inhibitory activity, probably attributed to the high phenolic content in these substances [182]. The methanolic and ethyl acetate extracts from the leaves showed powerful antioxidant properties comparable to known synthetic antioxidants which may be due to the constituents such as total flavonoids, total phenols, anthocyanins, chlorophyll, and carotenoid contents. In addition, both extracts showed moderate inhibitory activity against AChE with the ethyl acetate extract being more potent [182]. Essential oils obtained from the leaves and flowers, consisting of large amounts of monoterpenenes and oxygenated sesquiterpenes, have also been discovered to have protection against free radicals and oxidative stress as well as inhibiting AChE. For both free radical scavenging and anticholinesterase activities, leaf essential oil is reported to be better than flower oil [182]. Therefore, various extracts from this plant could potentially be used for the prevention of AD.

Several types of gall extracts of *Pistacia integerrima* have shown radical scavenging and cholinesterase inhibitory activity in vitro, which indicated that ethyl acetate extract exhibited the best radical scavenging activity and most potent AChE and butyrylcholinesterase inhibitory activities. Crude extract also demonstrated both AChE inhibitory and radical scavenging activities similar to those of the ethyl acetate extract. Free radical scavenging activity has been confirmed, with the two pure compounds isolated from this plant, quercetin and pyrogallol, as possessing high antioxidant activity especially for pyrogallol [192]. The in vivo study of the ethyl acetate and methanolic extracts of *P. integerrima* fruit together with four terebinth coffees revealed that both extracts showed moderate inhibitory activity towards butyrylcholinesterase but not acetylcholinesterase. They also exhibited radical scavenging activity at high concentrations. It has been revealed that terebinth coffee brands, with higher phenolic and flavonoid contents, possessed higher antioxidant and neuroprotective activities. The increased phenolic and flavonoid content may come from the roasting process, which suggests the roasting process of fruit as being a potential approach to improve the antioxidant properties of the extracts [182]. Similar to the other two plants in the same family, *P. integerrima* also has neuroprotective potential with mostly preventive effects for AD.

#### 3.1.2. Panax Ginseng

Ginseng has been widely used in eastern countries as one of the main representatives of traditional medicine and presents a variety of pharmacological actions. Recent studies on the efficacy of *Panax ginseng* extract against AD have demonstrated that it could inhibit the neurotoxicity induced by Aβ in vitro and attenuate Aβ accumulation in vivo in the brain of AD animal models [193]. In a mouse AD model, the fermented ginseng extracts reduced Aβ formation in the brain and improved memory function [194]. Through inhibiting AChE, the extracts from white, red, and black ginseng were able to protect the memory dysfunction in mice caused by hippocampal Aβ oligomer injection [195]. Ginseng extract, when given orally to the mice injected with Aβ oligomer, restored the decreased synaptophysin and ChAT activity [196]. *Panax ginseng* extract has been evaluated in a number of clinical trials on AD patients. Red ginseng extract, administered long-term with AD drugs, gradually improved cognitive functions with minor side effects on AD patients [197]. Red ginseng extract treatment for more than 12 weeks was approved to improve the frontal cortical activity in elderly AD patients [198]. Hundreds of reports have been published for the effectiveness of various ginseng extracts including white ginseng, red ginseng, and fermented ginseng on the alleviation of AD symptoms in animal models and patients. It is indicated that certain biologically active chemicals in the ginseng extracts may prevent cognitive dysfunction by reducing Aβ formation and aggregation. Those bioactive chemicals are mainly ginsenosides and gintonin, which have been found to protect from Aβ-induced neurotoxicity and reactive oxidative stress, stimulate the formation of sAPPα instead of Aβ, exhibit anti-inflammatory function, as well as enhance cholinergic systems, hippocampal neurogenesis, and cognitive functions [199]. It is believed that the neuroprotective effects from ginseng can be used for the prevention and treatment of AD, an idea that is being evaluated clinically.

#### 3.1.3. Phyllanthus Genus

The methanolic extract of Phyllanthus acidus (MEPA) exhibited neuroprotective effect through the improvement of cognitive functions and reduced oxidative stress via elevating the level of brain antioxidant enzymes as well as reducing lipid peroxidation and AChE activity. Thus, this plant extract can be useful in AD treatment [200]. To test the effects of Phyllanthus amarus and Cynodon dactylon in ameliorating AD-induced oxidative stress, the methanolic extracts of both plants were evaluated in an AD rat model. The results indicated that both extracts significantly increased the levels of superoxide dismutase, catalase, and NADH dehydrogenase compared to the control group. These antioxidant properties of the two herbal medicines may provide a new approach for AD treatment [201]. The ethanolic extracts of Phyllanthus emblica ripe and unripe fruits demonstrated marked beneficial effects on an AD mice model by improving the learning, memory, and antioxidant potential as well as decreasing AChE activity [202]. All the observed neuroprotective effects from *P. acidus* are both preventive and therapeutic for AD.

#### 3.1.4. *Ginkgo biloba* L. (Ginkgoaceae)

Its leaf extract (EGb) has been well known for the capacity to improve memory and age-related deterioration, and thus has been widely used in dietary supplements. This neuroprotective effect may be associated with the ability to scavenge free radicals, prevent mitochondrial dysfunction, activate JNK and ERK pathways, and inhibit neuronal apoptosis [203]. Co-administration of EGb and donepezil exhibited better anti-amnestic effect through more augmented pro-cholinergic and antioxidative effects of both drugs in a scopolamine-induced AD rat model without any change in their systemic/brain exposure [204]. Numerous clinical trials have been carried out on EGb for single dose studies and long-term studies, with mixed results. Analysis of both positive and negative results revealed that EGb could improve the cognitive function in AD patients with mild dementia with long-term administration and appropriate dosage [205]. The phytochemicals of EGb that provide neuroprotective activity are believed to be flavonoids, organic acids, and terpenoids [206]. It is widely accepted that *G. biloba* possesses neuroprotective effects which can be both preventive and therapeutic for AD.

#### 3.1.5. *Hibiscus sabdariffa* L. (Malvaceae)

As one of the most famous traditionally used remedies worldwide, it presents numerous pharmacological activities including sedative, antioxidant, anti-inflammatory, antidepressant, antiproliferative, antimicrobial, and neuroprotective activities. The neuroprotective effects of anthocyanin-enriched extracts from two Hibiscus varieties, white and red calyces, were tested in vitro to reveal their antioxidant potential and acetylcholinesterase inhibition activity. In vivo studies have indicated that Hibiscus extracts prevented memory impairment through the amelioration of STZ-induced neuroinflammation and amyloidogenesis. In summary, Hibiscus represents a promising safe preventive agent for AD with antioxidant, anti-inflammatory, anti-acetylcholinesterase, and anti-amyloidogenic activities. The LC/MS/MS analysis for the two Hibiscus extracts identified anthocyanins, flavonoids, and aliphatic and phenolic acids as being the major components responsible for the neuroprotective activity [207].

#### 3.1.6. *Hedera nepalensis* K. (Araliaceae)

Treatment of crude extract (HNC) on the AD rat model showed an increase in the levels of catalase (CAT) and superoxide dismutase (SOD) while reducing glutathione (GSH) levels. Significantly elevated levels of dopamine and serotonin identified in the midbrain region and decreases in cognitive and memory impairment in the treatment group supported this plant as being a potential therapeutic agent for AD and diabetes [208].

#### 3.1.7. *Salvia miltiorrhiza* B. (Lamiaceae)

As a widely used traditional Chinese medicine, the neuroprotective effects of this herbal medicine for AD treatment were reviewed along with its mechanisms and bioactive components [209]. Its extract protected SH-SY5Y cells against Aβ_25–35_-induced neurotoxicity through the inhibition of oxidative stress and the mitochondria-dependent apoptotic pathway [210]. A standardized fraction of *S. miltiorrhiza*, PF2401-SF, inhibited iNOS expression and NO production and exhibited anti-inflammatory activity on LPS-activated RAW 264.7 macrophages [211]. *S. miltiorrhiza* has also been found to efficiently induce neuron cell differentiation from rat mesenchymal stem cells [212]. While induced pluripotent stem cells (iPSCs) have the potential to differentiate into neural lineages, *S. miltiorrhiza* could promote the differentiation potential in vitro, and in vivo enhanced the survival and neural differentiation of transplanted iPSCs-derived neurons [213]. Its active chemical components, such as cryptotanshinone, tanshinone I, tanshinone IIA, Sal B, Sal A, and danshensu, have shown multiple neuroprotective effects including anti-Aβ, antioxidant, anti-apoptosis, anti-inflammation, enhancing cholinergic signaling, and inducing neurogenesis, which support the use of this plant as a preventive agent for AD [209].

#### 3.1.8. *Nardostachys jatamansi* D. (Caprifoliaceae)

Its ethanolic extract was examined in vitro and in vivo for its neuroprotective effect. It was found that the extract, along with major component chlorogenic acid, could inhibit Aβ-induced cell death in vitro. In an in vivo study using a Drosophila AD model, this extract was able to rescue the neurological phenotypes of Aβ_42_-expressing flies as well as prevent Aβ_42_-induced cell death in the brain. Other neuroprotective effects of this extract may come from the reduced number of glial cells, decreased level of ROS, NO, and ERK phosphorylation in Aβ_42_-expressing flies without changing Aβ accumulation. Thus, as a promising herbal medicine for AD treatment, *N. jatamansi* exerts its neuroprotective activity most likely via the combination of its antioxidant and anti-neuroinflammatory properties together with the inhibitory activity towards ERK signaling [214]. Another in vivo screening program with Drosophila AD models also identified *N. jatamansi* as having neuroprotective effects against Aβ_42_ neurotoxicity [215]. All observed neuroprotective effects from this plant are both preventive and therapeutic for AD.

#### 3.1.9. *Viscum album* L. (Santalaceae)

While disrupted BDNF levels have been indicated in AD pathogenesis, chronic treatment with *V. album* extract was found to significantly increase the BDNF levels in serum and diminish AlCl_3_-induced neurotoxicity in vitro and in vivo, which implied the neuroprotective effects of this plant which could be used as a preventive agent for AD [216].

#### 3.1.10. *Bacopa monnieri* L. (Plantaginaceae)

As a commonly used traditional medicine, this plant contains a significant number of bioactive components including saponins and triterpenoids as main compounds together with alkaloids, sterols, and polyphenols which are famous for their antioxidant activity [217]. It was used traditionally to improve memory and cognitive function [218]. In an AD rat model, *B. monnieri* extract decreased cholinergic degeneration and showed cognition-enhancing effect [219]. Another study revealed that AChE could be inhibited by *B. monnieri,* which resulted in increased ACh levels [220]. Its extracts were also found to protect neuronal cells from β-amyloid-induced damage by lowering ROS levels [221]. A clinical trial demonstrated that the polyherbal formulation containing *B. monnieri* extract could improve the cognitive functions efficiently through decreasing the inflammatory level and oxidative stress in patients [222]. In summary, this neuroprotective plant could be used as a preventive and therapeutic agent for AD.

#### 3.1.11. *Convolvulus pluricaulis* C. (Convolvulaceae)

The traditional medicine *C. pluricaulis* has been used for nervous system-related diseases such as stress, anxiety, mental fatigue, and insomnia [223]. Its ethanolic extract and the ethyl acetate and water fractions were found to significantly enhance learning and memory in rats [224]. Oral administration of *C. pluricaulis* alleviated the scopolamine-induced neurotoxic effect through decreasing tau and APP expression in the brain in AD rat models [225]. The active compounds that have been isolated from this plant including triterpenoids, flavanol glycosides, anthocyanins, and steroids, which are believed to provide the nootropic and memory-enhancing activity of the traditional medicine, making this plant a potential preventive agent for AD [226].

#### 3.1.12. *Centella asiatica* L. (Apiaceae)

This herbal medicine is used traditionally for rejuvenating the neuronal cells and for increasing intelligence, longevity, and memory [227]. In a mice AD model, its extracts were able to reduce the β-amyloid pathology and oxidative stress in the brain [228]. Its ethanolic extracts were also reported to protect neurons against the neurotoxicity induced by Aβ_1–40_, decrease ROS production, and activate the antioxidative defense system by increasing the activities of various related enzymes and enhancing levels of glutathione and glutathione disulfide [229]. All these activities indicate the great potential of this traditional medicine for AD prevention and treatment [230]. The major bioactive compounds identified from *C. asiatica* are asiatic acid and asiaticoside, which exert antioxidant activity in vitro to reduce cytotoxicity induced by H_2_O_2_, decrease the levels of free radicals, and prevent the cell damage caused by Aβ accumulation [230,231].

#### 3.1.13. *Uncaria rhynchophylla* M. (Rubiaceae)

As a medicinal herb used in the traditional Chinese medicine, *U. rhynchophylla* extract showed free radical scavenging activity and inhibited lipid peroxidation in an excitotoxicity animal model [232]. It has also been reported to exert protection against neuronal damage by reducing microglial activation, nNOS, iNOS, and apoptosis [233]. Moreover, it was found to inhibit Aβ fibril formation and also dissemble preformed Aβ fibrils in an AD model induced by Aβ_1–40_ and Aβ_1–42_ [234]. Bioactive compounds existing in *U. rhynchophylla* extract are mainly alkaloids such as rhynchophylline, isorhynchophylline, hirsutine, hirsuteine, corynanthine, corynoxine, and dihydrocorynantheine [235,236], among which rhynchophylline and isorhynchophylline are the most intensively studied and have been widely accepted as neuroprotective compounds [237]. All these neuroprotective effects suggest that this plant could be used for AD prevention and treatment.

#### 3.1.14. *Glycyrrhiza inflata* B. (Fabaceae)

As one of the Glycyrrhiza species, *G. inflata* is known to increase mitochondrial biogenesis and decrease oxidative stress [238]. In AD cell models, its aqueous extract displayed a significant reduction in ROS and tau misfolding with low levels of cytotoxicity [154]. Another in vitro study found that *G. inflata* extract and its two constituents licochalcone A and liquiritigenin demonstrated potent anti-Aβ aggregation and radical-scavenging activities. Moreover, both *G. inflata* extract and its constituents suppressed the production of NO, TNFα, IL-1β, PGE2, and/or Iba1 in LPS-stimulated RAW 264.7 or BV-2 cells and further protected cell death. Taken together, *G. inflata* extract, licochalcone A, and liquiritigenin exhibit neuroprotective functions through anti-oxidative and anti-inflammatory activities to prevent neuronal apoptosis and could be used as preventive and therapeutic agents for AD [239].

#### 3.1.15. Alpinia Oxyphylla-Schisandra Chinensis Herb Pair (ASHP)

The ethanolic extract of ASHP, together with its bioactive components, namely schisandrin (SCH) and nootkatone (NKT), was evaluated in the AD mice model. The results showed that in the object recognition task both the extract and SCH/NKT had a higher discrimination index with decreased levels of TNF-α, IL-1β, and IL-6. Thus, the neuroinflammation response was attenuated in both cases through the inhibition of the TLR4/NF-κB/NLRP3 pathway. Additionally, both treatments significantly restored the activities of glutathione S-transferase (GST), COX-2, SOD, total antioxidant capacity, and iNOS, as well as increased the levels of NO, GSH, and malondialdehyde. Both treatments were able to noticeably improve the histopathological changes of the hippocampus. Collectively, ASHP and its bioactive components exhibited neuroprotective effects by improving cognitive disorders, inhibiting the inflammatory reaction, and preventing oxidative stress, supporting their potential in AD prevention [240].

#### 3.1.16. *Buchanania axillaris* D. (Anacardiaceae), *Hemidesmus indicus* L. (Apocynaceae) and *Rhus mysorensis* G. (Anacardiaceae)

The methanolic extracts of these three medicinal plants have been found to be most active against type II diabetes (T2D) and AD. Their derived fractions were tested in vitro for their inhibitory capacities against cholinesterases and α- & β-glucosidase, as well as their antioxidant potency in scavenging radicals. The results revealed that all the methanolic extracts of the test plants could inhibit AChE, BuChE, and α- and β-glucosidase in a dose-dependent manner. Evaluation of the subsequent fractionation indicated that chloroform fractions for all three extracts were most potent in inhibiting all four enzymes. These active chloroform fractions also showed great neuroprotective effects against the cell death-induced oxidative stress while not affecting cell viability. Overall, these three medicinal plants, with their methanolic extracts and the derived chloroform fractions having strong anticholinesterase, antiglucosidase, antioxidant, and neuroprotective activities, could be a multifunctional therapeutic regime for T2D and AD [241].

#### 3.1.17. *Coriandrum sativum* L. (Apiaceae), *N. Jatamansi*, *Polygonum multiflorum* T. (Polygonaceae), *Rehmannia glutinosa* G. (Plantaginaceae), and *Sorbus commixta* H. (Rosaceae)

From a rapid in vivo screening platform using Drosophila AD models, these five medicinal plants out of 23 tested plants have been identified as exerting neuroprotective effects against Aβ_42_ neurotoxicity. Further investigation on the ethanolic extracts from *P. multiflorum* and *S. commixta* showed strong suppression of the AD neurological phenotypes. In vitro studies revealed that both ethanolic extracts increased the viability of Aβ-treated cells [215]. All observed effects support their utility in AD prevention and treatment.

#### 3.1.18. Bojungikgi-Tang (BJIGT; Bu Zhong Yi Qi Tang in China, Hochuekkito in Japan)

This traditional oriental herbal formula consisting of eight medicinal herbs was found to be effective in treating dementia in a clinical study in South Korea [242]. Its neuroprotective effect was assessed in vitro and in vivo. The results indicated that BJIGT inhibited Aβ aggregation and enhanced BACE activity in vivo and antioxidant activity in vitro. It also exerted neuroprotective effects against H_2_O_2_-induced damage in vitro and remarkably ameliorated cognitive impairments in vivo. Additionally, it could prevent the aggregation and expression of Aβ peptides, as well as the expression of NeuN and BDNF in the hippocampi of Aβ-injected mice. Therefore, BJIGT may have great potential as a therapeutic option for treatment of AD and dementia [242].

#### 3.1.19. Fuzhisan (FZS)

As a Chinese herbal complex which contains *Scutellaria baicalensis *G.** (Labiatae), Ginseng root (Araliaceae), *Glycyrrhiza uralensis *F.** (Leguminosae), and *Anemone altaica *F.** (Araceae) [243], FZS has been clinically used for senile dementia for more than fifteen years [244]. Studies have indicated that FZS increased cognitive function in AD animal models [243] and AD patients [245]. Its neuroprotective effects are found to be associated with anti-apoptosis and anti-Aβ accumulation activities, as well as enhancing ACh levels and neurotrophic effects [246]. A recent study has also indicated that the neuroprotective function of FZS may protect against Aβ-induced neurotoxicity. All evidence implied that FZS may be clinically beneficial for AD patients.

### 3.2. Neuroprotective Natural Products from Food for AD

Some neuroprotective natural products, especially the antioxidant natural products, are naturally occurring in the human diet including fruits, vegetables, nuts, seeds, flowers, and some herbal beverages. It is of interest to summarize these food-related natural products for their neuroprotective properties.

#### 3.2.1. *Momordica charantia* L. (Cucurbitaceae)

The fruits of four strains were dried and ground to give four mixtures, MC2, MC3, MC5, and MC5523, which were investigated in vitro and in vivo for their pharmacological functions in the treatment of AD. Neuroprotective effects have been observed for all four fruit powders while MC5523, in combination with LiCl, was found to increase the survival rate as well as enhance neuroprotection associated with anti-gliosis in an AD mice model. Moreover, MC5523 and LiCl cotreatment prevented memory deficits via reduced gliosis, oligomeric Aβ level, tau hyperphosphorylation, and neuronal loss while increasing the expression levels of synaptic-related protein and pS9-GSK3b, which provided a potential strategy for AD treatment [247]. Another study investigated the ethanolic extract of its fruits on memory impairment in an AD mice model and found that the ethanolic extracts could improve the anti-amnesic activity in mice via the inhibition of lipid peroxidation and the decreased AChE activity in the brain [248]. Most of the neuroprotective effects associated with *M. chrantia* are preventive for AD.

#### 3.2.2. *Benincasa hispida* L. (Cucurbitaceae)

The aqueous extract of BH pulp was administered orally to AD mice before the intracerebroventricular infusion of colchicine (bilaterally), preventing SP formation. This neuroprotective effect might be attributed to the chemical constituents containing vitamins A, C, and E, flavanols, and flavonoids, which possess antioxidant activity. Thus, it was concluded that *B. hispida*, with its preventive potential for AD through antioxidant scavenging actions, protected rat neurons from the damage caused by colchicine-induced oxidative stress through the prevention of dentate granule cell destruction in the hippocampus and through preventing the extracellular deposition of senile plaques in a colchicine-induced AD rat model [249].

#### 3.2.3. *Allium sativum* L. (Alliaceae)

The neuroprotective effects of aged garlic extract (AGE) have been well documented including antioxidant, anti-neuroinflammatory, and regulatory effects on neurotransmitter signaling in the brain that may be associated with the pathogenesis of AD [250,251]. A recent study examining the effect of AGE on Aβ_1–42_-induced cognitive dysfunction and neuroinflammation in an AD rat model demonstrated that in cognitively impaired rats, AGE could remarkably improve short-term recognition memory. Moreover, it reduced the activation of microglia and IL-1β level to substantially minimize the inflammatory response [252]. Low temperature-aged garlic extract has been discovered to suppress psychological stress through the modulation of stress hormones and oxidative stress response in the brain [253]. All observed effects from AGE are preventive for AD.

#### 3.2.4. *Curcuma longa* L. (Zingiberaceae)

The active components in this spice are curcuminoids such as curcumin and related compounds [254]. Curcumin has been reported to have various interesting activities including anti-inflammatory, antioxidant, antitumor, and antibacterial activities [255]. Its potential in AD treatment has been reviewed before [256]. A recent review with a total of 32 studies that focused on curcumin’s effect on in vitro and in vivo AD models approved that curcumin may be a promising approach for AD [257]. Its ethanolic extract has been found to exert neuroprotective activity through antioxidant effects [258]. Another study on the herbal extract approved its neuroprotective effect as it was able to attenuate CeCl_3_-induced oxidative stress, enhance the activities of antioxidant enzymes, and decrease AChE activity [259]. It is hypothesized that people using turmeric frequently may have a lower incidence of AD, indicating turmeric’s preventive effect [260]. Studies have revealed a possible link between regular intake of turmeric as part of curry and excellent cognitive performance among the elderly [261]. For AD patients, turmeric treatment could also greatly improve their behavioral symptoms, supporting the therapeutic potential of turmeric for AD treatment [262].

#### 3.2.5. *Zingiber officinale* R. (Zingiberaceae)

Widely used as extracts or as ingredients of ginger tea in food supplements, *Z. officinale* showed AChE inhibitory activity in vitro. It possesses the capacity to inhibit lipid peroxidation and exerts a neuroprotective effect against AD. In vivo studies on AD rat models with extract from *Z. officinale* have reported reduced lipid peroxidation levels. The neuroprotective mechanism associated with *Z. officinale* extract may be attributed to its ability to reduce the overstimulated NMDA receptors and prevent the production of free radicals [263], which may suggest the preventive and therapeutic potential of this plant for AD. The principal chemical components identified from *Z. officinale* include gingerols, shogaols, bisabolene, zingiberene, and monoterpenes [264].

#### 3.2.6. *Punica granatum* (Pomegranate)

The juice and extracts from pomegranate fruit have been reported to possess neuroprotective effects against AD pathogenesis in various animal models [265,266,267]. Its neuroprotective mechanisms may involve counteracting oxidative stress [267], reducing brain inflammation [266], and decreasing accumulation of soluble Aβ_42_ and amyloid deposition in the hippocampus [265]. A study on the bioactive chemical components of pomegranate implied that the relevant brain-absorbable compounds responsible for the anti-AD effects of pomegranate were urolithins which showed protective effects on Aβ_42-_induced neurotoxicity and paralysis [268]. The observed neuroprotective effects are both preventive and therapeutic for AD.

#### 3.2.7. *Oryza sativa* (Rice Berry)

Oxidative stress is well known to be associated with AD pathogenesis. The study of the rice berry, which is rich in antioxidant components, on an AD rat model revealed that it could significantly protect against memory impairment and neurodegeneration in the hippocampus. The hippocampal AChE activity and the lipid peroxidation products were also decreased, which suggested the rice berry as a potentially effective agent for AD prevention and treatment [269].

#### 3.2.8. *Vitis vinifera* L. (Grape) and Red Wine

As a major component in the Mediterranean diet, the benefit of regular intake of red wine at moderate amounts for lowering AD and dementia development has been systematically reviewed and it is suggested that low to moderate wine drinking could reduce the risk of dementia and AD by at least a third, indicating its AD preventive role [270]. Evidence has been provided that extracts from grape skin and grape seed can inhibit Aβ aggregation, presenting an AD treatment potential [271]. In an AD rat model, red grape juice intake has been found to increase learning speed and improve memory [272]. Grape seed proanthocyanidins presented the capacity to ameliorate neuronal oxidative damage and cognitive impairment in an experimental AD model [273]. In vitro and in vivo studies on grape seed proanthocyanidins proved that they may be a novel therapeutic strategy for AD treatment [274]. Grape leaves’ polyphenolic extract has shown neuroprotective function through its antioxidative, anti-neuroinflammatory, and anti-amnesic activities against AlCl_3_-induced cerebral damages and neurocognitive dysfunction [275]. The bioactive components in grapes and red wine are believed to be polyphenols which are well established as antioxidants, with resveratrol being the most intensively studied for its neuroprotective effects.

#### 3.2.9. Nuts Including Almond, Hazelnut, and Walnut

All of these nuts, considered as beneficial for the brain, exert neuroprotective effects that contribute to alertness, concentration, and memory [276]. Many studies have proven that nuts contain a rich matrix of bioactive chemicals and could possess the capacity to support neuronal function in the brain. The relation between nut consumption, improved cognitive performance, and lowered incidence of AD has been confirmed by several studies, supporting their AD preventive properties [277]. Adding hazelnut kernel into rats’ diet led to enhanced memory, reduced anxiety, and ameliorated neuroinflammation and apoptosis. As a dietary supplement, hazelnut has been confirmed to support healthy aging [278]. Almond supplementation in rats’ diet for one to two weeks significantly reversed amnesia induced by scopolamine. This effect was believed to have resulted from reduced AChE activity as well as lowered cholesterol and triglyceride levels, together with slightly increased glucose level [279]. Adding almond paste for 4 weeks in rats’ diet significantly improved learning and memory with enhanced brain tryptophan monoamine levels and serotonergic turnover in the brain [280]. Significantly improved memory retention was also observed in another study with 4 weeks of almond administration to rats, which was attributed to the elevated level of Ach [281]. Almond and walnut supplementation for 4 weeks attenuated cadmium-induced memory impairment in rats, possibly through cholinergic and antioxidant activities [282]. A walnut enriched diet has been shown to improve cognitive and motor performance [283]. Its neuroprotective effects are believed to be associated with the remarkably attenuated expression of proinflammatory cytokines, decreased level of AChE, significantly restored levels of antioxidant enzymes, and reduced expression of NF-κB [284]. Walnut could also increase the number of Ach receptors and upregulate expression of ChAT [285]. In rat PC12 cells, the peptides from defatted walnut protein exhibited significant free radical scavenging and cytoprotective activities against oxidative damage [286]. Walnut extract inhibited Aβ fibril formation and reduced Aβ-mediated cell death [287]. All observed neuroprotective effects associated with nuts are preventive for AD.

### 3.3. Neuroprotective Natural Products from Marine Sources for AD

Marine natural products, developed under adverse conditions and containing unusual structures, have been a rich source for the treatment of numerous diseases including AD. A number of marine natural products, together with their derived synthetic analogs, showed good efficacy against AD [288]. Thus, marine natural products are actively sought after as neuroprotective agents for AD.

#### 3.3.1. Marine Macroalgae (Seaweeds)

Marine macroalgae which are plant-like organisms normally found in coastal areas contain a wide variety of bioactive chemicals such as polyphenols, polysaccharides, pigments, amino acids, peptides, and proteins. The health benefits associated with marine macroalgae and their bioactive compounds have been summarized in several excellent review papers [289]. The methanolic extract of *Eisenia bicyclis* (Kjellman) Setchell, a perennial brown seaweed, showed neuroprotective activity together with its ethyl acetate and n-butanol subfractions by reducing intracellular ROS production in PC12 cells induced with Aβ_25–35_. The components that contributed to this activity were believed to be phlorotannins eckol, phlorofucofuroeckol A, and 7-phloroeckol [290]. Another brown seaweed, *Ishige foliacea* has been found to contain a phlorotannin-rich fraction that improved memory impairment in mice through the additive or synergistic effect from several mechanisms including reducing brain AChE activity, suppressing oxidative stress, and activating the ERK-BDNF-CREB signaling pathway [291]. All these studies indicated that marine macroalgae present great options for AD prevention and treatment through protecting neuron damage and improving memory impairment by multiple pathways to exhibit neuroprotective activity which may result from the effects of its bioactive components and the additive or synergistic effects of those bioactive components.

#### 3.3.2. Spirulina Cyanobacteria

Mostly referred to as blue-green algae, *Cyanobacteria* are actually prokaryotic organisms more closely related to bacteria with interesting pharmacological properties [292]. *Spirulina platensis* is a cyanobacterium known to have abundant nutritive elements such as carotenoids, polysaccharides, polyunsaturated fatty acids, vitamins, minerals, and protein [293]. It has been reported that *S. platensis* protein extract was a potent antioxidant which was able to scavenge free radicals with its chelating capacity and prevent radical-mediated cell death in vitro [294]. A follow-up study revealed that the aqueous extract of *S. platensis* and its active component C-phycocyanin could reduce cytotoxicity and inhibit the expression of inflammation-related genes like COX-2, TNF-α, IL-6, and iNOS in vitro, suggesting its AD preventive effects [295]. Another cyanobacterium, *Spirulina maxima*, contains many physiologically active chemicals including carotenoids, polysaccharides, chlorophylls, C-phycocyanin, and vitamins [296]. Its extract was found to ameliorate learning and memory impairments in an AD mice model, implicating its potential in AD treatment. This neuroprotective effect was believed to come from decreased expression levels of hippocampal Aβ_1–42_, APP, and BACE1, as well as decreased AChE activity, suppressed hippocampal oxidative stress, increased BDNF level, and activated BDNF/PI3K/Akt signaling pathways [297]. In another study with PC12 cells treated with Aβ_1–42_, the *Spirulina maxima* extract prevented Aβ-induced oxidative stress and cell death through the activation of BDNF signaling [298].

#### 3.3.3. Thalassospira Profundimaris

A screening program with two hundred and twenty-five marine bacterial extracts looking at both their toxicity and neuroprotective properties identified several marine bacterial extracts as promising leads, with *Thalassospira profundimaris* being the most potent one. Its crude extract was able to preserve synaptic structure in vitro. An in vivo follow-up study revealed its AD preventive capacity because it could block the cell cycle-related neuron death. While none of the fractions derived from the crude extract exhibited neuroprotective activity as potent as the whole extract, it was hypothesized that the synergistic action of several components might be responsible for the overall effects [299].

All the neuroprotective natural products discussed above have been summarized below in Table 1.

## 4. Problems and Concerns with Natural Products for AD

While natural products and their isolated natural compounds have been well established as neuroprotective agents and valuable resources for exploring novel approaches in AD treatment, many of them still remain untested and their clinical use is not easy to monitor for a wide variety of reasons [300]. First of all, the quality of the natural source materials for natural products depends not only on genetic factors, but also on other extrinsic factors including environmental conditions, harvest time, and agricultural and collection practices for the source materials, making it difficult to perform quality control on the raw materials. The general requirements and methods for quality control of the finished mixture of natural products that contain hundreds of natural constituents are even more complex.

Although several of the above-mentioned natural product extracts or mixtures such as *P. ginseng*, EGb, *B. monnieri*, AGE, and *C. longa* have been evaluated in various stages of clinical trials, not all of them exhibited remarkable therapeutic effects in AD patients. However, they could still be used for the prevention of AD. Some specific limitations and challenges associated with natural products and their isolated natural compounds that might affect their clinical efficacy in AD treatment are their physicochemical instability, their limited water solubility, their rapid metabolism, their low bioavailability, and their distribution to the CNS, which have been adequately summarized in several reviews [301,302,303,304]. Several neuroprotective natural compounds, especially the polyphenols like resveratrol and curcumin, are chemically unstable and can be easily degraded or converted to inactive derivatives [303]. The presence of the blood-brain barrier requires enough lipophilicity from the natural products to be able to penetrate the CNS, which may create extra obstacles for some neuroprotective natural products, including polyphenols and polysaccharides, and limit their clinical efficacy. Carotenoids and alkaloids, on the other hand, are lipophilic enough to cross the blood-brain barrier, but their poor water solubility brings other problems which lead to low bioavailability. It has also proved very challenging to translate the exciting preclinical results of the neuroprotective natural products to clinical applications. Although several natural product mixtures like EGb and ginger extracts have been evaluated in AD patients, no conclusive positive results have been obtained.

To improve the bioavailability of the neuroprotective natural products useful in AD treatment, nanotechnology and nanocarrier-based strategies have been developed in the delivery of natural product mixtures and the isolated bioactive compounds which may enhance the therapeutic response and improve clinical efficacy [305,306]. The most commonly used nanoparticles include polymeric nanoparticles, solid lipid nanoparticle, crystal nanoparticle, nanogels, liposomes, micelles, and dendrimer complexes. Several studies have described the incorporation of nanoparticle delivery systems for natural products and their bioactive compounds. One noteworthy example is the nanolipidic EGCG particles, which have been approved to almost double the neuronal α-secretase enhancing ability in vitro and improve EGCG’s oral bioavailability in vivo by more than two-fold [127].

## 5. Conclusions

Mounting evidence has demonstrated the great neuroprotective potentials of natural products and natural bioactive compounds in AD treatment with few harmful side effects. Although not fully understood, the pathological process associated with AD is believed to be multifactorial. Neuroprotective strategies involving multiple mechanisms of action are important for the prevention and treatment of AD. Natural product mixtures or extracts, with multiple bioactive compounds and the ability to exert multiple neuroprotective mechanisms, are preferable in AD drug discovery. With more practical and comprehensive quality control guidelines developed to ensure the safety and efficacy of natural product therapies, as well as new approaches and strategies to help promote the CNS access of these neuroprotective agents, such as the incorporation of nanotechnology in the delivery of natural products, natural product therapy could play an essential role in the prevention and treatment of AD.

## Figures and Tables

**Table 1:** Status of Neuroprotective Natural Products Extracts and Mixtures.

Natural Products	Extract	Neuroprotective Effects Found	Research Status	References
*Pistacia vera*	Kernel	Inhibited cisplatin or vincristine-induced cognitive and motor impairments	In vivo, rats	[183]
*Pistacia lentiscus*	Essential oil	Attenuated lipopolysaccharide-induced memory impairment and decreased AChE activity and oxidative stress markers in brain tissue	Rats	[187]
*Pistacia integerrima*	Gall extracts	Radical scavenging and cholinesterase inhibitory activity	In vitro	[192]
*Pistacia atlantica*	Ethyl acetate and aqueous extracts	AChE inhibitory activity	In vitro	[182]
*Panax ginseng*	Root extracts	Reduced Aβ formation, inhibited AChE, restored the decreased synaptophysin and ChAT activity, reduced Aβ formation and aggregation	In vitro, mice and clinical trials	[193,194,195,196,197,198,199]
*Phyllanthus acidus*	Methanolic extract	Improvement of cognitive functions and reduced oxidative stress via elevating the level of brain antioxidant enzymes, as well as reducing lipid peroxidation and AChE activity	In vitro	[200]
*Phyllanthus amarus, Cynodon dactylon*	Methanolic Extract	Increased the levels of superoxide dismutase, catalase, and NADH dehydrogenase	Rats	[201]
*Phyllanthus emblica*	Ethanolic extract	Improved learning, memory, and antioxidant potential, as well as decreased AChE activity	Mice	[202]
*Ginkgo biloba* L.	Leaf extract (EGb)	Scavenged free radicals, prevented mitochondrial dysfunction, activated JNK and ERK pathways, and inhibited neuronal apoptosis	Scopolamine-induced AD rat model, clinical trials	[203,204,205]
*Hibiscus sabdariffa* L.	Anthocyanin-enriched extracts	Prevented memory impairment through the amelioration of STZ-induced neuroinflammation and amyloidogenesis	In vitro, mice	[207]
*Hedera nepalensis* K.	Crude extract	Increased the levels of catalase (CAT) and superoxide dismutase (SOD), while reducing glutathione (GSH) levels	Rats	[208]
*Salvia miltiorrhiza* B.	Root extract	Inhibited oxidative stress and the mitochondria-dependent apoptotic pathway. Inhibited iNOS expression and NO production. Induced neuron cell differentiation from rat mesenchymal stem cells. Promote the differentiation potential of iPSCs and enhanced the survival and neural differentiation of transplanted iPSCs-derived neurons.	In vitro, rat	[210,211,212,213]
*Nardostachys jatamansi* D.	Ethanolic extract	Inhibited Aβ-induced cell death	In vitro, *Drosophila* model	[214,215]
*Viscum album* L.	Extract	Significantly increased BDNF levels in the serum and diminished AlCl_3_-induced neurotoxicity	In vitro, mice	[216]
*Bacopa monnieri* L.	Extract	Decreased cholinergic degeneration and showed cognition-enhancing effects, protect neuronal cells from β-amyloid-induced damages by lowering ROS levels, inhibited AChE	Rats, clinical trials	[217,218,219,220,221,222]
*Convolvulus pluricaulis* C.	Ethanolic extract	Decreased tau and AβPP expression in the brain	Rats	[223,224,225,226]
*Centella asiatica* L.	Ethanolic extract	Reduced β-amyloid pathology and oxidative stress in the brain; protected neurons against the neurotoxicity induced by Aβ_1–40_, decreased ROS production, and activated the antioxidative defense system by increasing the activities of various related enzymes and enhancing levels of glutathione and glutathione disulfide	Mice	[227,228,229,230,231]
*Uncaria rhynchophylla* M.	Root extract	Showed free radical scavenging activity and inhibited lipid peroxidation; reduced microglial activation, nNOS, iNOS, and apoptosis; inhibited Aβ fibril formation and also dissemble preformed Aβ fibrils	Rats	[232,233,234,235,236,237]
*Glycyrrhiza inflata* B.	Aqueous extract	Reduction in ROS and tau misfolding, potent anti-Aβ aggregation and radical-scavenging activities. Suppressed the production of NO, TNFα, IL-1β, PGE2, and/or Iba1	In vitro	[154,238,239]
*Alpinia oxyphylla-Schisandra chinensis herb pair (ASHP)*	Ethanolic extract	Inhibition of the TLR4/NF-kB/NLRP3 pathway; restored the activities of GST, COX-2, SOD, total antioxidant capacity, and iNOS, increased the levels of NO, GSH, and malondialdehyde	Mice	[240]
*Buchanania axillaris* D., *Hemidesmus indicus* L. and *Rhus mysorensis* G.	Methanolic extracts	Inhibit AChE, BuChE, and α- and β-glucosidase, neuroprotective effects against the cell death-induced oxidative stress	In vitro	[241]
*Coriandrum sativum* L., *N. jatamansi*, *Polygonum multiflorum* T., *Rehmannia glutinosa* G., and *Sorbus commixta* H.	Ethanolic extract	Neuroprotective effects against Aβ_42_ neurotoxicity	Drosophila AD models	[215]
*Bojungikgi-tang (BJIGT)*	Herbal formula	Inhibited Ab aggregation, enhanced BACE activity in vivo, and increased antioxidant activity; prevent the aggregation and expression of Aβ peptides, NeuN and BDNF in the hippocampus	In vitro, mice, clinical trials	[242]
*Fuzhisan (FZS)*	Herbal complex	Anti-apoptosis and anti-Aβ accumulation activity, enhancing ACh levels, and neurotrophic effects	Rats, Clinical tirals	[243,244,245,246]
*Momordica charantia* L.	Dried and ground fruit	Reduced gliosis, oligomeric Aβ level, tau hyperphosphorylation, and neuronal loss, Increasing the expression levels of synaptic-related protein and pS9-GSK3b	In vitro, rats	[247,248]
*Benincasa hispida* L.	Aqueous extract	Prevented SP formation; antioxidant scavenging actions; prevention of dentate granule cell destruction in the hippocampus and by preventing the extracellular SP deposition	Rats	[249]
*Allium sativum* L.	Aged garlic extract	Reduced the activation of microglia and IL-1 level, minimized the inflammatory response; suppressed psychological stress through modulation of stress hormones and oxidative stress response in the brain	Rats	[250,251,252,253]
*Curcuma longa* L.	Ethanolic extract	Attenuate CeCl_3_-induced oxidative stress, enhanced the activities of antioxidant enzymes, and decreased AChE activity	In vitro, mice, rats, clinical trials	[254,255,256,257,258,259,260,261,262]
*Zingiber officinale* R.	Root extract	Inhibited AChE, inhibited lipid peroxidation; reduced the overstimulation of NMDA receptors and prevented the production of free radicals	Rats	[263,264]
*Punica granatum*	Juice and extracts	Counteraced oxidative stress, reduced brain inflammation, decreased the accumulation of soluble Aβ_42_, and reduced amyloid deposition in the hippocampus	Mice	[265,266,267,268]
*Oryza sativa*	Dietary supplement	Decreased hippocampal AChE activity and lipid peroxidation products	Rats	[269]
*Vitis vinifera* L.	Juice, polyphenolic extract	Inhibited Aβ aggregation; antioxidative, anti-neuroinflammatory, and anti-amnesic activities	Rats	[270,271,272,273,274,275]
Hazelnut (*Corylus avellana*)	Kernel	Enhanced memory, reduced anxiety, and ameliorated neuroinflammation and apoptosis	Rats	[276,278,279]
Almond (*Prunus dulcis*)	Paste	Reduced AChE activity, lowered cholesterol and triglyceride levels; improved learning and memory with enhanced brain tryptophan monoamine levels and serotonergic turnover	Rats	[279,280,281,282]
Walnut (*Juglans regia*)	Defatted protein	Attenuated expression of proinflammatory cytokines, decreased level of AChE, significantly restored levels of antioxidant enzymes, and reduced expression of NF-κB	Rats	[283,284,285,286,287]
*Eisenia bicyclis*	Methanolic extract	Reduced intracellular ROS production in PC12 cells induced with Aβ25–35	In vitro	[290]
*Ishige foliacea*	Phlorotannin-rich fraction	Reducing brain AChE activity, suppressed oxidative stress, and activated the ERK-BDNF-CREB signaling pathway	Mice	[291]
*Spirulina platensis*	Protein and aqueous extracts	Scavenged free radicals and prevented radical-mediated cell death; reduced cytotoxicity and inhibited the expression of inflammation-related genes like COX-2, TNF-α, IL-6, and iNOS	In vitro	[293,294,295]
*Spirulina maxima*	Ethanolic Extract	Decreased expression levels of hippocampal Aβ_1–42_, APP, and BACE1 decreased AChE activity, suppressed hippocampal oxidative stress, increased BDNF level, and activated BDNF/PI3K/Akt signaling pathways	Mice	[296,297,298]
*Thalassospira profundimaris*	Crude extract	Preserved synaptic structure; blocked cell cycle-related neuron death	In vitro, mice	[299]

## Abbreviations

AD	Alzheimer’s Disease
Aβ	amyloid β peptide
NFT	neurofibrillary tangle
ROS	reactive oxygen species
RNS	reactive nitrogen species
Nrf2	nuclear-factor erythroid 2-related factor
ARE	antioxidant response element
NF-κΒ	nuclear factor κΒ
MAPK	mitogen-activated protein kinase
IL-6	interleukin 6
LPS	lipopolysaccharide
EGCG	epigallocatechin-3-gallate
ERK	extracellular signal-regulated kinase
APP	amyloid precursor protein
SP	senile plaques
AICD	APP intracellular domain
BACE	beta-site amyloid precursor protein cleaving enzyme
CTF-β	carboxy-terminal fragment beta
PP2A	protein phosphatase 2A
CDK-5	cyclin-dependent kinase 5
GSK-3	glycogen synthase kinase-3
ACh	acetylcholine
AChE	acetylcholinesterase
ChAT	choline acetyltransferase
BDNF	brain-derived neurotrophic factor
PKC	protein kinase C
NGF	nerve growth factor
PPAR-α	peroxisome proliferator-activated receptor alpha
AChT	acetylcholine transferase
sAPPa	soluble amyloid precursor protein a
MEPA	methanolic extract of Phyllanthus acidus
EGb	*Ginkgo biloba*
CAT	catalase
SOD	superoxide dismutase
GSH	glutathione
GST	glutathione S-transferase
BuChE	butyrylcholinesterase
T2D	type II diabetes
AGE	aged garlic extract
